# Diagnosis, Prevalence and Significance of Obesity in a Cohort of CKD Patients

**DOI:** 10.3390/metabo13020196

**Published:** 2023-01-28

**Authors:** Diego Moriconi, Claudia D’Alessandro, Domenico Giannese, Vincenzo Panichi, Adamasco Cupisti

**Affiliations:** Department of Clinical and Experimental Medicine, University of Pisa, 56126 Pisa, Italy

**Keywords:** obesity, body mass index, fatty mass, adiposity, anthropometry, bio-impedance, CKD

## Abstract

Background: data regarding the association between obesity and morbidity/mortality in patients with chronic kidney disease (CKD) are uncertain and sometimes contradictory. The aims of our study were to determine the associations among different measures of obesity and adiposity, and the risk of all-cause mortality or dialysis entry in stage 3–5 CKD patients. Materials: this observational cohort study included 178 CKD patients followed for a median of 71 months. Biochemistry, anthropometric measures such as body mass index (BMI), waist-to-hip ratio, mid-arm muscle circumference (MAMC) and body composition by bioimpedance analysis were evaluated. Results: we found a weak agreement between BMI and other measures of adiposity. In multivariable regression analysis, all measures of obesity such as BMI, waist circumference and waist-to-height ratio were not associated with dialysis entry and/or mortality. Instead, MAMC was associated with dialysis entry HR 0.82 [95% CI: 0.75–0.89] and high FM% with mortality HR 2.08 [95% CI: 1.04–4.18]. Conclusions: in our CKD population, lower MAMC was predictive of dialysis commencing, while a higher percentage of fatty mass was a predictor of mortality. Instead, obesity, as defined by BMI, is not associated with dialysis commencing or all-cause mortality.

## 1. Introduction

Obesity is a pandemic that is rapidly increasing worldwide, and it frequently coexists with other diseases such as diabetes, hypertension and chronic kidney disease (CKD) [1,2].

Despite the fact that obesity is believed to be strongly associated with the development and progression of CKD, observational studies have reported contradictory findings regarding the association between obesity and morbidity/mortality in renal patients.

Previous studies of individuals with CKD reported a J-shaped association between obesity, as defined by body mass index (BMI), and mortality, with a higher risk of death observed in underweight and severely obese categories compared with normal-weight individuals [3,4].

However, observational studies conducted in hemodialysis patients showed a protective role of obesity in cardiovascular and all-cause mortality [5,6].

Data on the paradoxical associations of obesity have become increasingly consistent over time. Recent studies have shown that a higher BMI is associated with better survival in the end stage kidney disease (ESKD) population [7,8]. A meta-analysis by Ladhani et al. [9] confirmed this association, observing a non-linear relationship between obesity and all-cause mortality in predialysis and hemodialysis patients, with the greatest risk of death observed for the extreme categories of BMI.

Another study confirmed the inverse relationship between BMI and mortality in hemodialysis patients, which remained consistent across different ethnicities after accounting for potential confounders [10].

However, a non-negligible potential limitation of these features in the CKD population may be the discrepancy between the definition of obesity, namely as abnormal or excessive fat accumulation by the World Health Organization (WHO), and the clinical measures of obesity, based on BMI values (WHO 2021).

In fact, although BMI is easy to calculate in clinical practice, it is an inaccurate indicator both of body composition and nutritional status, because BMI does not differentiate between fluids, muscle and fat mass [11], and it does not reflect body fat distribution between subcutaneous and visceral deposits [12].

It is now well-established that visceral fat is more metabolically active and provides a greater contribution to the inflammatory milieu associated with metabolic syndrome, endothelial dysfunction and kidney or cardiovascular damage, when compared to subcutaneous fat [13,14,15]. Therefore, reliance on BMI may misclassify patients; consider those with excess adiposity but low muscle mass, that may incorrectly be considered as non-obese or individuals with large muscle mass and good fat distribution who could be diagnosed as obese.

Moreover, being overweight and obesity are largely prevalent in the CKD population. In the more advanced stages of CKD, the indication exists that protein restriction must never be coupled with a low-energy intake. This is the reason why the question sometimes arises about which dietary approach must be implemented in obese CKD patients.

The aims of our study were to determine the agreement among different measures of obesity, and the risk of all-cause mortality or ESKD in individuals affected by stage G3–G5 CKD that are not on dialysis.

## 2. Materials and Methods

### 2.1. Participants and Study Design

One hundred and seventy-three outpatients (27 females, 146 males) aged 66 ± 12 years affected by CKD stage 3–5 not on dialysis, who were metabolically and nutritionally stable, entered the study during the period November 2017–October 2019.

Patients with severe heart failure (Stage IV NYHA), respiratory insufficiency, cancer, dementia, psychiatric or neurologic diseases, inflammatory systemic diseases, with a Barthel Index and/or Karnofsky score <100 or those who did not give their consent to the study were excluded [16]. All recruited patients underwent nutritional and functional assessment to assess body composition: this included biochemistry, anthropometry, bioimpedance analysis and functional tests.

After baseline evaluation, patients were followed up for 65 ± 21 months. The main outcomes were mortality for all causes or dialysis commencing.

### 2.2. Biochemistry

Biochemistry included serum levels of creatinine, blood urea nitrogen (BUN), phosphorus, calcium, albumin, bicarbonate, parathyroid hormone (PTH) and hemoglobin. Tests were performed using standard laboratory methods. Glomerular filtration rate (eGFR) was estimated using the CKD-EPI formula [17].

### 2.3. Anthropometry

Body weight was assessed on a mechanical scale with the patient wearing light clothes and no shoes. Height was measured with a stadiometer. Body mass index (BMI) was calculated as body weight/height^2^ (Kg/m^2^). Waist circumference, hip circumferences and mid-arm circumference (MAC) were measured at the nearest cm with a flexible narrow. Triceps skinfold thickness (TST) was measured by a caliper at the middle-third of the non-dominant arm. Mid-arm muscle circumference (MAMC) was calculated from MAC and TST using a standard formula: MAMC = MAC − (3.14 × TST).

### 2.4. Body Composition Analysis

Body composition analysis was estimated using a Bioelectrical Impedance single frequency Analyzer (BIA/STA, Akern, Florence, Italy) with a distal, tetrapolar technique, delivering an excitation current at 50 kHz. Impedance (Z) represents the force that interferes with the flow of electric current and is given by the vectorial sum of the resistance (Rz) and the reactance (Xc), the two bioelectric parameters given by the body analyzer. The phase angle is the most immediate bioelectric index resulting from a proportion between resistance and reactance according to this formula: phase angle = Arctang (Xc/Rz) * 180 * π. Body cell mass (BCM) is derived from bio-impedance analysis and body cell mass index (BCMI) is consequently calculated, as well as fatty mass (FM) and the percentage of fatty mass (FM% = FM/weight * 100) [18].

### 2.5. Central Obesity Measures

A cut-off point of waist circumference > 102 cm for males and >88 cm for females was chosen to identify patients at increased cardio-metabolic risk [19], identifying patients with very high cardiovascular mortality (very high-risk waist).

Waist-to-height ratio (WtHR) was defined as the waist circumference (as cm) divided by the height (as cm); a cut-off point of 0.59 was chosen to identify patients with abdominal obesity in both genders, in accordance with previous studies [20,21].

Finally, we set the upper limits of fatty mass (FM) for defining obesity as FM > 25% in males and >35% in females with age <60 years, and with FM > 30% in males and >42% in females with age >60 years. These values correspond to a BMI of 30 kg/m^2^ in Caucasians [22,23] and they were defined as High% FM.

### 2.6. FM%/MAMC Ratio

The FM% and MAMC were calculated as previously described and expressed as a percentage of the expected reference values, based on the Third National Health and Nutrition Examination Survey, (NHANES) III, adjusted for gender and age [24].

The formula (FM%/FM% std)/(MAMC/MAMC std) was applied to calculate the FM%/MAMC ratio, as a potential new adimensional parameter of adiposity and reduced appendicular skeletal mass.

### 2.7. Statistical Analysis

Variables were tested for normality using the Shapiro–Wilk Test. Continuous variables are reported as the mean ± standard deviation, or median [interquartile range]. Categorical data were described as count (percentage). Group differences based on the BMI cut-off were compared using the χ^2^ test for categorical variables and the Mann–Whitney U test for continuous variables.

The composite outcome was defined as either starting of renal replacement therapy (dialysis) or death from any cause during follow-up. Cohen’s kappa coefficient was used to measure inter-rater reliability between the actual definition of obesity based on BMI cut-off and the other anthropometric measures of central obesity (based on percentage of fatty mass, waist and WtHR). Multivariable Cox proportional hazards models were applied for risk factor analysis of the three main outcomes: (a) mortality, (b) dialysis and (c) composite outcome, accounting for potential confounders, and *p* value was calculated by Wald test. Progression-free survival curves were realized by Kaplan–Meier method and log-rank test was applied to evaluate the differences between the curves. Differences were considered as statistically significant when *p* < 0.05. All data processing was carried out by JMP Pro 15.3.0 (SAS Institute Inc., Cary, NC, USA)

## 3. Results

### 3.1. Anthropometric and Clinical Features

The study participants were divided into three groups based on their BMI, expressed as kg/m^2^: BMI ≥ 30 (30.7%), 30 > BMI ≥ 25 (46.8%) and BMI < 25 (22.5%). It was found that the majority of CKD patients (77.5%) were overweight or obese, and none of the patients were underweight (BMI < 18.5 kg/m^2^).

The BMI ≥ 30 group had a higher prevalence of type-2-diabetes (*p* = 0.007) and higher fasting blood glucose levels (*p* = 0.01) compared to the other two groups. Further, there were no differences among groups as regards the other haematochemical parameters analyzed such as eGFR, creatinine clearance, lipidic profile, albumin and electrolytes (Table 1).

As expected, the BMI > 30 group had a significantly larger waist circumference, WHR and WtHR compared to the other groups. Additionally, triceps skinfold thickness was also higher in the BMI ≥ 30 group, while no differences were detected between the 30 > BMI ≥ 25 and BMI < 25 groups. Instead, MAC and MAMC progressively increased from the BMI < 25 group to the BMI > 30 group, while TBW% was progressively decreased (Table 1).

### 3.2. Agreement between the Main Measures of Obesity

The prevalence of Very High-Risk Waist was 98.1%, 66.7% and 12.8 % in BMI ≥ 30, 30 > BMI ≥ 25 and BMI < 25 groups, respectively (*p* < 0.0001). All the subjects in the BMI ≥ 30 group had a WtHR > 0.59, which was also prevalent in more than one half (64.2%) of the patients in the 30 > BMI ≥ 25 group; however, it was prevalent in only 5.1% of the BMI < 25 group (*p* < 0.0001). The presence of High% FM was 77.1%, 70.4% and 39.5% in the BMI ≥ 30, 30 > BMI ≥ 25 and BMI < 25 groups, respectively (*P* < 0.0001) (Table 1).

Finally, the study found a weak agreement between the definition of obesity based on BMI (BMI ≥ 30 kg/m^2^) and the other measures of central obesity, such as Very High-Risk Waist, WtHR > 0.59, High% FM (K Cohen coefficient 0.37, 0.44 and 0.32, respectively) (Table 2).

### 3.3. Predictors of Outcome

After a median follow-up of 71 months (range 3–98 months), the start of replacement therapy and all-cause mortality occurred, respectively, in 41 (24%) and 45 (26%) patients, whereas the composite outcome occurred in 78 patients (45%).

**Figure 1:**
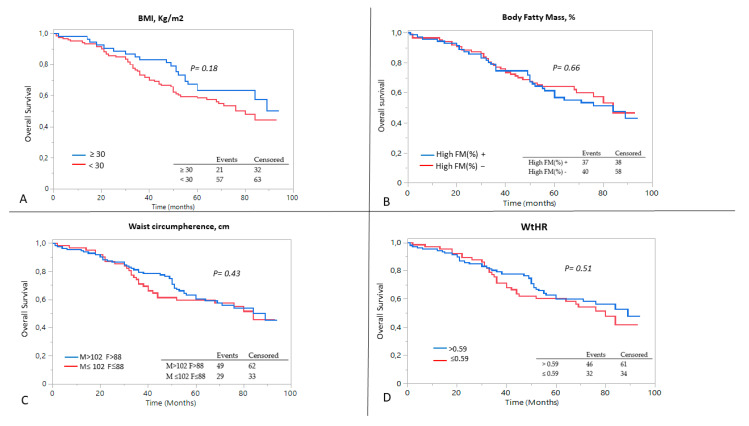
Kaplan–Meier curves for the composite outcome of ESKD and mortality, after dividing the patients on the basis of (**A**) BMI, (**B**) percentage of body fat mass, (**C**) waist circumference and (**D**) waist-to-height ratio. A log-rank analysis was conducted.

Figure 1 illustrates the Kaplan–Meier survival analysis of patients based on all of the measures of obesity and adiposity, including BMI > 30 kg/m^2^, WtHR > 0.59, High %FM and Very-High cardiovascular risk Waist. The analysis showed that there were no differences in the event rate of the composite outcome in obese patients compared to non-obese patients, regardless of the parameter considered.

**Figure 2:**
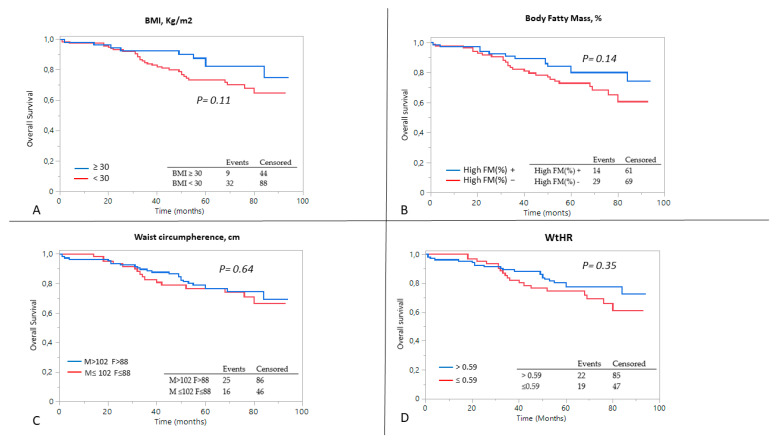
Kaplan–Meier curves for kidney outcome (ESKD) after dividing the patients on the basis of (**A**) BMI, (**B**) percentage of body fat mass, (**C**) waist circumference and (**D**) waist-to-height ratio. A log-rank analysis was conducted.

**Figure 3:**
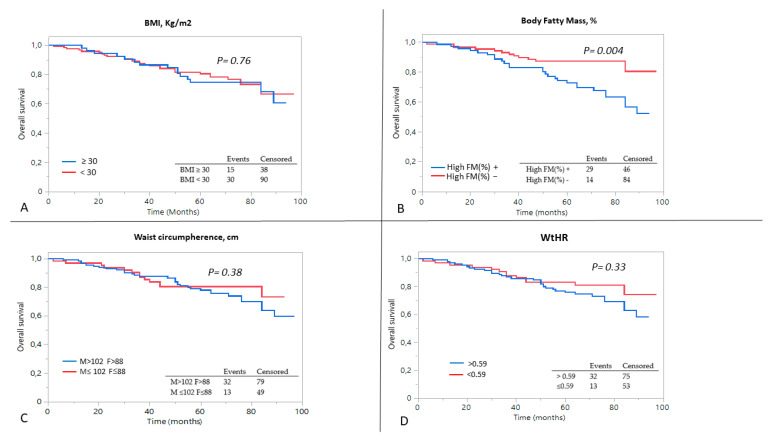
Kaplan–Meier curves for the mortality outcome, after dividing the patients on the basis of (**A**) BMI, (**B**) percentage of body fat mass, (**C**) waist circumference and (**D**) waist-to-height ratio. A log-rank analysis was conducted.

Similarly, none of the parameters related to obesity (BMI > 30 kg/m^2^, WtHR > 0.59, High %FM and Very-High Risk Waist) had an impact on the cumulative probability of starting dialysis (Figure 2). In terms of all-cause mortality, after dividing the population by the percentage of fatty mass, the event rate was higher in subjects with High %FM compared to patients with fatty mass within the normal range (log-rank test 0.001, Figure 3).

Using the Cox proportional hazard model, adjusting for the main confounders (Table 3), the risk of the all-cause mortality increased with lower baseline eGFR, HR 0.96 [95% CI: 0.94–0.99], and with High FM%, HR 2.08 [95% CI: 1.04–4.18].

A more marked reduction in renal function at baseline correlated with a higher rate of dialysis entry; as well, it occurred for phosphorus serum level HR 3.50 [95% CI: 1.95–6.41], while larger values of MAC, HR 0.85 [95% CI: 0.77–0.93] and of MAMC, HR 0.82, [95% CI: 0.75–0.89] were associated with a better outcome.

Nevertheless, in addition to baseline residual kidney function, only MAMC, HR 0.89 [95% CI: 0.83–0.96], MAC, HR 0.89 [95% CI: 0.84–0.95], and phosphorus serum level, HR 2.29 [95% CI: 1.49–2.55], were shown to be independent predictors of a composite outcome (Table 3).

### 3.4. FM%/MAMC

The distribution of FM%/MAMC is reported in Table 1. In te male gender it was higher than in females (1.17 ± 0.27 vs. 0.95 ± 0.26, respectively, *p* = 0.002). In univariate analysis there was no correlation with eGFR, while there was a positive correlation with age (R= 0.27, *p* = 0.002) and BMI (R = 0.34, *p* < 0.0001). In the multivariable Cox regression model, FM%/MAMC showed a very significant association with mortality, HR 3.63 [95% CI: 1.07–11.47] (Table 3).

## 4. Discussion

The findings of the present cohort study allow for drawing some interesting considerations. There is a lack of correlation between the traditional definition of obesity, based on BMI cut-off values, and anthropometric measures of central obesity/adiposity or body composition obtained through bio-impedance analysis.

In CKD patients, obesity defined as BMI ≥ 30 kg/m^2^ is not associated with negative outcomes such as an increased risk of ESKD or all-cause mortality. However, a higher percentage of fatty mass, a more specific measure of adiposity and a higher FM% to MAMC ratio (which may be also an indicator of sarcopenia) were linked to all-cause mortality in CKD populations.

Taken as a whole, our study reinforces the understanding that obesity is a common comorbidity in patients affected by CKD, with 78% of our cohort being overweight or obese. Additionally, our findings are consistent with most of the previous research, which shows that, in pre-dialysis patients, being overweight and obesity assessed by BMI do not have a negative impact on the main outcomes. However, BMI is not paradoxically associated with greater survival, unlike results that have been reported by other studies on CKD [25]. One of the main differences between the present and other published studies, which can explain the imperfect agreement in the protective role of obesity on mortality, may be the absence in our cohort of underweight patients, as assessed by body mass index <18.5 kg/m^2^.

In fact, many studies have identified a J-shaped association between BMI and mortality in general populations [26,27], and being underweight has been associated with an increased risk of all-cause and cardio-vascular mortality in CKD patients [28,29]. In this setting, low body weight may be due to malnutrition, which is a major risk for mortality, therefore configuring a reverse-causation bias [30].

This may be the reason why a non-negligible prevalence of underweight subjects could highlight a paradoxically protective effect of obesity on mortality which is not evident in our study.

Nevertheless, our study offers a new perspective on chronic kidney disease by suggesting that certain measures of central obesity and adiposity may not be protective but, rather, may increase the mortality.

Specifically, a high percentage of fatty mass was found to be strongly associated with a higher mortality rate in our cohort of CKD patients, highlighting the limitations of using BMI alone to accurately assess body composition.

In the present study, about 60% of overweight patients have central obesity on the basis of the WtHR [31]. Additionally, a large percentage (70%) of these patients would be classified as obese based on their level of adiposity, expressed as fatty mass percentage (FM%). An accurate measurement of body composition in patients with CKD is clinically relevant because of the high prevalence of protein-energy wasting and change in adiposity distribution observed in the CKD population [32]. Hence, in daily clinical practice, a more precise body composition assessment should be mandatory in order to reduce the risk of a misclassification.

A reduction in muscle mass together with increased body fat could be the expression of sarcopenic obesity [33], which represents a strong marker for a worse clinical prognosis in general populations [34]. In the present study, we confirm that a marker of adiposity and reduced somatic muscle mass such as FM%/MAMC is also a good predictor of mortality in CKD patients.

The MAMC is an anthropometric parameter that is well related to the appendicular skeletal muscle mass, which is not confounded by visceral fatty free mass. In the present study, MAMC, but not free-fatty mass, was associated with dialysis entry. In other words, patients with greater skeletal muscle mass had a lower risk of dialysis initiation.

Based on this assumption, we believe that the use of a ratio between the FM%, which progressively increases with BMI [35], and the MAMC could be a reliable indicator to identify patients with an unfavorable body composition characterized by a high proportion of fat mass and low somatic protein reserves.

In accordance with our results, in a pooled analysis involving seven prospective studies in the general population, a J-shaped association between fat mass and mortality was found, with a 50% increased mortality risk observed for a high versus low level of fatty mass measured by bioimpedance analysis. Furthermore, among patients aged ≥65 years, the authors demonstrated both a strong linear positive relation between fatty mass, and an inverse relationship between lean mass and mortality [36]. Our data suggest that the same observations could be extended to our CKD population, and that the obesity paradox may not be evident when measures that are more specifically characterized body composition are taken into account.

However, there are conflicting results in the literature regarding the prognostic role of body composition distribution in CKD. Muscle and fat mass abnormalities were examined in participants of the National Health and Nutrition Examination Survey 1999–2004. A reduction in appendicular skeletal muscle mass index (ASMI) <5.45 kg/m^2^ in women and <7.26 kg/m^2^ in men resulted as an independent risk factor for increased mortality in the normal-weight subclass. However, it was not associated with a higher mortality risk in CKD patients [37]. On the contrary, a longitudinal study including 287 CKD patients reported an increased mortality rate among those who had a reduction in handgrip strength and diminished MAMC [38].

Unfortunately, most studies in the general population are heterogeneous in terms of design, follow-up duration and eGFR level, and few studies have examined the sarcopenic-obese phenotype directly [39]: these are some aspects that can explain the non-univocal results obtained.

In our opinion, several pathogenetic hypotheses exist which explain the association of both adiposity and reduction in muscle mass with mortality in patients with CKD.

It is well known that an excess of adipose tissues stimulates the release of inflammatory mediators such as cytokines and tumor necrosis factor α, causing a pro-inflammatory milieu and oxidative stress [40]. A similar persistent low-grade inflammation has also been recognized as an important component of CKD, due to increased production and decreased clearance of pro-inflammatory cytokines, intestinal dysbiosis and metabolic acidosis [41].

Regardless of the cause, chronic inflammation leads to muscle wasting via accelerated muscle protein breakdown [1], and this could affect survival through several mechanisms. In fact, better-preserved muscle mass could help maintain functional status and increase energy stores, improving metabolism and reducing the frailty and hospitalization linked to sarcopenia, which can contribute to a higher mortality risk [42,43]. Furthermore, chronic inflammation can cause a breakdown of immune tolerance, leading to major alterations in tissues and organs, which can increase the risk for many non-communicable diseases that collectively represent the leading causes of mortality worldwide [44]. All these mechanisms could explain the association between adiposity, sarcopenia and a higher mortality rate [45].

Another consideration that derives from our data concerns the nutritional approach to CKD obese patients. It is known that a low-protein diet without energy restriction is beneficial in terms of reducing the nitrogen requirement and maintaining muscle mass and nutritional status [46].

Hence, a conflict exists in obese CKD patients, namely as to whether to prescribe a low-energy normal protein diet or low-protein normal energy diet.

Our data show that, once CKD is present, obesity or being overweight based on BMI do not negatively impact renal replacement therapy entry, while a high percentage of fatty mass and a greater skeletal appendicular muscle component are associated with increased mortality. These findings could suggest that physical activity may be important for advanced stages of CKD, in addition to a low-protein diet without energy restrictions.

In fact, it has been observed that a low-protein diet can reduce uremic toxins and inflammation, and improve metabolic acidosis. These mechanisms affect anabolic intracellular signals that promote protein degradation, leading to muscle mass loss [47]. Additionally, physical activity, may be a method to increase and maintain lean muscle mass while reducing fat mass, preserving the appendicular skeletal mass and muscle strength [48].

In obese individuals, more than one hour of daily moderate-intensity aerobic exercise induced weight loss similarly to dietary restrictions alone, with reductions in abdominal subcutaneous and visceral fat; furthermore, even in the case of no weight loss, regular exercise could reduce subcutaneous and visceral fat, ameliorating body composition quality.

In advanced stages of CKD, a tailored exercise regimen can effectively modify body composition and decrease both mortality and the likelihood of starting dialysis. Additionally, when the physical activity comes to dietary intervention for obese CKD patients, an accurate assessment of energy needs is crucial in order to prevent both excessive energy intake and sarcopenia. For this reason, an objective measurement of energy expenditure should be encouraged using indirect calorimetry to measure resting energy expenditure plus an objective evaluation of physical activity with accelerometers [49].

Our study suggests that a comprehensive approach that includes a correct assessment of energy expenditure and energy requirements, a tailored physical activity program and a regular monitoring of body composition may be an effective method to address being overweight and obesity in CKD patients, while also addressing metabolic abnormalities and delaying the need for dialysis through protein restriction. Of consequence, it is conceivable that, in obese CKD, a low protein, normal energy diet may be implemented, combining adequate physical activity in order to mostly obtain a favorable balance between fat and free-fat mass, and correct uremic abnormalities.

Several limitations of our study should be noted. First of all, the number of participants was relatively small, thus limiting the ability to adjust for a high number of covariates in multivariate analysis. For example, the limited number of patients with BMI > 30 kg/m^2^ does not make it possible to evaluate the strength of the association between mortality and predictors of sarcopenia and adiposity separately in obese vs non-obese subjects.

Secondly, this is a single-center cohort study including people of the same ethnicity, while several studies have pointed out the discrepancy between race/ethnicity across sarcopenia indices [50]. This could mean that our results are not universally generalizable.

Furthermore, it is well established that, in observational real-life studies, the imbalances in covariates between groups could lead to systematic biases.

Nonetheless, this study had also some strengths, such as the detailed characterization of the population on the basis of measures of adiposity and central obesity and the relatively long-term follow up.

## 5. Conclusions

In conclusion, this observational cohort study showed poor agreement between the current definition of obesity based on BMI cut-off values and the definition of central obesity or adiposity based on anthropometric or instrumental measurements. In addition, obesity, as defined by BMI, is not associated with an increased risk of ESKD or all-cause mortality. Instead, a higher percentage of fatty mass, and a higher FM% to MAMC ratio, were associated with all-cause mortality in CKD populations.

Taken as a whole, our study confirmed the evidence that, once CKD develops, definitions of being overweight and obesity based on BMI do not impact pre-dialytic conditions in a composite outcome, as shown in previous studies; however, at the same time, we would place an emphasis on the need for a better definition of adiposity/central obesity, which appear to be associated with a worse outcome in terms of mortality.

## Figures and Tables

**Table 1:** Baseline features of patients consecutively enrolled in the study. Anthropometric and laboratory values are reported as mean ± SD, median [IRQ] or n (%).

	BMI ≥ 30	30 > BMI ≥ 25	BMI < 25	*p*-Value
Subjects, n (%)	53 (30.7)	81 (46.8)	39 (22.5)	
Age, yrs	68 ± 9	71 ± 10 *	66 ± 16	0.037
Gender, F%	9 (17.0)	11 (13.6)	7 (18.0)	0.781
Weight, kg	92 ± 11	77 ± 8	67 ± 8	<0.0001
BMI, Kg/m^2^	32.5 ± 2.3	27.3 ± 1.3	23.2 ± 2.4	<0.0001
Diabetes, n (%)	23 (43.4)	15 (18.5)	10 (25.6)	0.007
Waist circumpherence, cm	111 ± 8	101 ± 8	90 ± 9	<0.0001
Hip circumpherence, cm	111 ± 7	104 ± 5	98 ± 4	<0.0001
WHR	1.01 ± 0.07	0.98 ± 0.08	0.91 ± 0.07	0.001
WtHR	0.66 ± 0.03	0.60 ± 0.04	0.53 ± 0.05	<0.0001
FM, %	33.8 ± 6.7	29.1 ± 5.9	22.4 ± 6.4	<0.0001
TBW (%)	58.3 ± 4.6	53.4 ± 4.3	50.9 ± 5.1	<0.0001
TST, mm	13 [11–20] ^§^	11 [9–16]	10 [7–14]	0.002
MAC, cm	32.6 ± 4.0	29.5 ± 2.3	27.2 ± 2.5	<0.0001
MAMC, cm	28 [26–30]	25 [24–27]	24 [22–25]	<0.0001
FM%/MAMC	1.21± 0.23	1.17± 0.27	0.98± 0.30 *	0.0002
Categorical variables related to obesity and adiposity				
Waist (>102 M or >88 F), n (%)	52 (98.1)	54 (66.7)	5 (12.8)	<0.0001
WtHR (>0.59), n (%)	53 (100.0)	52 (64.2)	2 (5.1)	<0.0001
High FM%, n (%)	41 (77.4)	31 (39.5)	5 (12.8)	<0.0001
Haematochemical features				
eGFR, mL/min/1.73	34 ± 12	30 ± 13	30 ± 12	0.222
S Creatinine, mg/dL	1.98 [1.57–2.76]	2.19 [1.65–2.99]	2.34 [2.00–2.81]	0.213
S Urea, mg/dL	77 [57–92]	82 [63–101]	68 [56–99]	0.286
S Total cholesterol, mg/dL	170 ± 37	177 ± 31	176 ± 36	0.632
S LDL cholesterol, mg/dL	99 ± 32	106 ± 31	106 ± 27	0.501
S Tryglicerides, mg/dL	121 [112–156]	119 [111–158]	118 [113–154]	0.522
S Glucose, mg/dL	105 [91–113] ^§^	94 [84–105]	90 [80–108]	0.013
S Albumin, g/dL	4.20 ± 0.31	4.15 ± 0.36	4.23 ± 0.52	0.732
S Potassium, mEq/L	4.7 ± 0.5	4.6 ± 0.5	4.7 ± 0.4	0.776
S Phosphorus, mg/dL	3.3 ± 0.6	3.3 ± 0.6	3.2 ± 0.6	0.509

Abbreviations: BMI; body mass index; FM%, body mass percentage; TBW (%); total body water percentage; WHR; waist to hip ratio; WtHR; waist to height ratio; TST; triceps skinfold thickness; MAC; middle arm circumference; MAMC; mid-arm muscle circumference. *p*-value < 0.05: * BMI < 25 vs. 30 > BMI ≥ 25. ^§^ BMI ≥ 30 vs. 30 > BMI ≥ 25. BMI ≥ 30 vs. BMI < 25.

**Table 2:** Reliability between the classical definition of obesity, based on BMI value, and the main measures of central obesity.

	Unweighted K Cohen	95% IC	*p* Value
Waist (>102 M or >88 F)	0.37	0.27–0.47	<0.0001
WtHR > 0.59	0.44	0.32–0.53	<0.0001
High FM%	0.40	0.27–0.55	<0.0001

Abbreviations: WtHR; waist to height ratio. High FM%: fatty mass (FM) >25% in males and >35% in females with age <60 years, and FM > 30% in males and >42% in females with age >60 years.

**Table 3:** Conditions associated with: (A) mortality, (B) dialysis and (C) composite outcome, in univariate and multivariable Cox regression model adjusted for baseline covariates (age, gender and presence of type 2 diabetes).

Factors	Univariate Analysis		Multivariate Analysis		
	HR (95% CI)	*p* Value	HR (95% CI)	Coefficient	*p* Value
**Mortality**					
eGFR, mL/min/1.73	0.96 (0.93–0.99)	0.005	0.96 (0.94–0.99)	−0.04	0.008
High% FM	2.61 (1.31–5.17)	0.001	2.08 (1.04–4.18)	−0.37	0.039
WHR	6.29 (1.28–51.35)	0.029	3.74 (0.36–39.59)	2.82	0.271
FM%/MAMC	6.97 (2.38–20.18)	0.0005	3.64 (1.07–11.47)	1.26	0.034
**Dialysis**					
eGFR, mL/min/1.73	0.88 (0.85–0.91)	<0.0001	0.87 (0.84–0.90)	−0.14	<0.0001
BMI, kg/m^2^	0.92 (0.85–0.99)	0.042	0.93 (0.85–1.01)	−0.08	0.079
Waist circumference, cm	0.97 (0.94–0.99)	0.001	0.97 (0.94–1.01)	−0.03	0.091
MAC, cm	0.87 (0.80–0.94)	0.0005	0.85 (0.77–0.93)	−0.17	0.0004
MAMC, cm	0.84 (0.78–0.91)	<0.0001	0.82 (0.75–0.89)	−0.20	<0.0001
S Phosphorus, mg/dL	3.30 (1.89–5.79)	<0.0001	3.50 (1.94–6.41)	1.25	<0.0001
**Composite outcome**					
eGFR, mL/min/1.73	0.92 (0.89–0.94)	<0.0001	0.92 (0.90–0.94)	−0.09	<0.0001
MAC, cm	0.89 (0.83–0.94)	0.0005	0.89 (0.84–0.95)	−0.12	0.001
MAMC, cm	0.88 (0.84–0.94)	0.0001	0.89 (0.83–0.96)	−0.12	0.0007
S Phosphorus, mg/dL	2.29 (1.51–3.45)	<0.0001	2.30 (1.49–3.55)	0.83	0.0002

Abbreviations: eGFR; estimated glomerular filtration rate; BMI; body mass index; FM%, body mass percentage; WHR; waist to hip ratio; MAC; middle arm circumference; MAMC; mid-arm muscle circumference; HR; hazard ratio; CI; confidence interval.

## Data Availability

The data that support the findings of this study are available from the corresponding authors upon reasonable request.
